# Absence of Association between Cord Specific Antibody Levels and Severe Respiratory Syncytial Virus (RSV) Disease in Early Infants: A Case Control Study from Coastal Kenya

**DOI:** 10.1371/journal.pone.0166706

**Published:** 2016-11-16

**Authors:** Joyce Uchi Nyiro, Charles Jumba Sande, Martin Mutunga, Patience Kerubo Kiyuka, Patrick Kioo Munywoki, John Anthony G. Scott, David James Nokes

**Affiliations:** 1 Kenya Medical Research Institute (KEMRI) -Wellcome Trust Research Programme, Centre for Geographic Medicine Research-Coast, Kilifi, Kenya; 2 London School of Hygiene and Tropical Medicine, London, United Kingdom; 3 School of Life Sciences and WIDER Centre, University of Warwick, Coventry, United Kingdom; 4 University of Oxford, Oxford, United Kingdom; Imperial College London, UNITED KINGDOM

## Abstract

**Background:**

The target group for severe respiratory syncytial virus (RSV) disease prevention is infants under 6 months of age. Vaccine boosting of antibody titres in pregnant mothers could protect these young infants from severe respiratory syncytial virus (RSV) associated disease. Quantifying protective levels of RSV-specific maternal antibody at birth would inform vaccine development.

**Methods:**

A case control study nested in a birth cohort (2002–07) was conducted in Kilifi, Kenya; where 30 hospitalised cases of RSV-associated severe disease were matched to 60 controls. Participants had a cord blood and 2 subsequent 3-monthly blood samples assayed for RSV-specific neutralising antibody by the plaque reduction neutralisation test (PRNT). Two sample paired t test and conditional logistic regression were used in analyses of log_2_PRNT titres.

**Results:**

The mean RSV log_2_PRNT titre at birth for cases and controls were not significantly different (P = 0.4) and remained so on age-stratification. Cord blood PRNT titres showed considerable overlap between cases and controls. The odds of RSV disease decreased with increase in log_2_PRNT cord blood titre. There was a 30% reduction in RSV disease per unit increase in log_2_PRNT titre (<3months age group) but not significant (P = 0.3).

**Conclusions:**

From this study, there is no strong evidence of protection by maternal RSV specific antibodies from severe RSV disease. Cord antibody levels show wide variation with considerable overlap between cases and controls. It is likely that, there are additional factors to specific PRNT antibody levels which determine susceptibility to severe RSV disease. In addition, higher levels of neutralizing antibody beyond the normal range may be required for protection; which it is hoped can be achieved by a maternal RSV vaccine.

## Introduction

Respiratory syncytial virus (RSV) is the most common viral cause of severe acute lower respiratory infections among children aged less than 5 years [1–3]. Global estimates indicate that RSV is present in 29% of all ALRI episodes[1] and responsible for approximately 66000–199000 deaths of under 5 year old children worldwide, with majority (99%) of these deaths occurring in developing countries[2].

A target group for RSV prevention is children under 6 months old who are highly susceptible to severe RSV associated disease[4]. Development of a vaccine to provide direct protection to the infant has been impeded by historical failure of a formalin inactivated vaccine[5, 6] and difficulties in designing an immunogenic live-attenuated vaccine that is well tolerated by the young infant [7]. An alternative approach currently under consideration as a means of protecting the vulnerable infant from RSV disease during the first few months of life is through vaccine boosting of maternal RSV-specific antibodies.

The concept of maternal vaccination has previously been used to prevent other viral infections such as poliovirus and influenza among infants or, in the case of rubella, in the foetus[8]. There is evidence that RSV specific maternal antibodies provide protection from severe RSV disease [9–11]. In addition, monthly repeated prophylaxis with Palivizumab^™^ (a humanized monoclonal IgG antibody) during the first RSV season among high risk infants reduced the risk of RSV associated hospitalization by 55% [12]. Studies have also shown that there is efficient transplacental transfer of RSV IgG antibodies during the third trimester of pregnancy, implying that maternal immunization has the potential to benefit the young infant most susceptible to RSV infections[13, 14]. The concept of boosting of maternal antibody following challenge is supported by significant population level rise in RSV-specific cord neutralising antibody in synchrony with seasonal RSV [15–17].

Maternal RSV vaccines under development are based on the fusion F protein which is a target of neutralising antibodies and known to be highly conserved between variants. Immunization with sub-unit RSV F protein has been shown to be safe and immunogenic in post-partum women [8, 18]. The leading candidate, an F protein nanoparticle design [19], (http://sites.path.org/vaccinedevelopment/respiratory-syncytial-virus-rsv/) is well tolerated and immunogenic in healthy adults and 3^rd^ trimester women (NCT02247726)) and is now undergoing phase 3 clinical trials (NCT02624947).

Despite these advances, the development of a maternal RSV vaccine is hindered by lack of quantitative data on the level of maternal RSV specific neutralising antibodies at birth required to provide protection against RSV disease among infants, and the duration over which this protection would last. Here we report results of a case control study from a birth cohort in Kilifi, a coastal part of Kenya, in which, the main objective was to quantify the level of RSV-specific maternal antibodies at birth that provide infant protection against severe disease. In this context, we asked the following questions (i) Is there a quantifiable level of antibody that provides protection in early life? (ii)Is the protective level absolute or is there a degree of protection against severe disease that varies with antibody level? (iii) Is there a differential rate of decay of maternal antibodies in those who get RSV disease against those who do not?

## Material and Methods

### Study site, Population and Design

This study was conducted in Kilifi, the coastal part of Kenya [20]. The study was nested within a previous birth cohort study. Between 1999 and 2007, Kenya Medical Research Institute-Wellcome Trust Research Programme (KEMRI-WTRP), conducted a Kilifi Birth Cohort (KBC) study within the Kilifi Health and Demographic Surveillance System [20–22]. The KBC study was an observational study where participants were followed for a two-year period with a cord blood sample collected at birth and subsequent 3 monthly blood samples during follow ups. Details of the birth cohort study are described elsewhere [16, 21, 22]. The KBC study participants had access to Kilifi County Hospital (KCH) previously referred to as Kilifi District Hospital (KDH).

We conducted a case-control study using archived serum or plasma samples from participants of the KBC study. We defined cases as infants who were admitted with severe pneumonia or lower respiratory tract infection (LRTI) within the first 6 months of life and had a positive RSV diagnosis by an Immunofluorescent Antibody Test (IFAT; Millipore, USA). Controls were infants who were not admitted to KCH with RSV associated severe pneumonia during the follow up period. Exposure to RSV infection among the controls was measured by screening cord blood and the 3 monthly subsequent samples for RSV IgA ELISA antibodies. An increase in RSV IgA antibodies detected either in the 3 or 6 month serum sample was used as a marker of exposure. Cases were matched to controls in a ratio of 1:2 by date of birth (within 30 days of date of birth) and geographical location. Every participant had a cord blood sample and two subsequent 3 months follow up blood samples. Continuous surveillance for RSV in paediatric admissions to KCH with syndromic severe or very severe pneumonia (using WHO criteria)[23] was in place throughout the KBC study with screening for RSV antigen by Immunofluorescent Antibody Test (IFAT)[24]. Enhanced detection of RSV cases missed by using the WHO pneumonia criteria was made by inclusion of children with a clinical admission diagnosis of LRTI. During the period for RSV surveillance, all paediatric admissions had blood samples collected for culture to diagnose invasive bacterial pathogens. A computerised system linking residents of the KHDSS and KCH admissions enabled identification of RSV positive hospital admission from the KBC.

Furthermore, we investigated whether the level of neutralizing antibodies measured from RSV A2 infection differs according to the circulating strain within the population. To do this, an additional subset of 100 cord blood samples was randomly selected from the Kilifi birth Cohort study regardless of infection status and tested for neutralizing activity to a range of RSV virus strains.

### Ethical Approval

All parents and guardians gave written consent to have their children participate in the KBC study or paediatric RSV study at KCH and for storage of blood samples for use in future research. The use of the archived sample set was approved by the KEMRI-Ethical Review Committee.

### Laboratory Procedures

Cord blood and admission samples collected during the KBC study were immediately taken to the microbiology laboratory for processing and storage at -80°C. For this case control study, archived blood samples were retrieved and assayed for RSV neutralising antibody by a plaque reduction neutralisation assay. Serum samples were incubated at 56°C in a water bath for thirty minutes to inactivate complement cascade proteins; thereafter, plaque reduction neutralisation procedures were conducted as described elsewhere[25]. The dilution (and titre reciprocal) of a test serum sample required to induce 50% neutralization of a known titration of RSV A2 virus was determined using the Spearman Karber method. To account for any significant effect of freeze thaw on sample neutralization titres, a validation assay was carried out where a set of KBC samples previously screened (5 years interval); were retrieved, screened and the two PRNT titres results compared.

A subset of 100 cord bloods collected over the same time period as for the case-control study were selected at random, stratified by year, from the KBC archive, as previously described [16]. These were screened for RSV specific neutralising antibodies by the method described above using 4 different strains i.e., RSV A2 (Australia, 1961), RSV B860 (Sweden, 1960), RSV A Kilifi (Kenya, 2005) and RSV B Kilifi (Kenya, 2005).

Residues of samples from controls were tested for RSV-specific IgA antibodies by ELISA using crude virus extract from laboratory adapted RSV A2 culture[26] Specific antibody concentrations were recorded as log arbitrary units (AU) as determined by a local standards procedure[26]. The crude virus RSV lysate preparation was as previously described by Ochola et al[27].

### Statistical analyses

All data analysis was conducted using STATA version 13.1 (College Station, Texas). Laboratory data for sample PRNT titres were logarithmically transformed (base 2) and merged with the KHDSS and clinical data for analyses.

To quantify the level of RSV-specific maternal antibodies at birth that provide infant protection, mean PRNT titres were computed for cord blood samples. The difference in cord blood levels between the cases and controls were analysed using a two sample paired t test. For comparison, a two sample Wilcoxon rank-sum (Mann-Whitney) test was applied to the log_2_PRNT titres for cases and controls. Further comparison of the distribution of cord blood log-transformed PRNT titres between cases and controls was done using reverse cumulative distribution plots. The absolute reduction in risk of RSV disease per unit increase in cord blood antibody titres was calculated using modified conditional logistic regression methods[28]. The estimated rate of decay of RSV specific log_2_PRNT titres from birth to 6 months of life was determined by simple linear regression, accounting for clustering of titres for samples from the same individual using the procedure for Huber-White sandwich estimator. Elimination of bias on the rate of decay arising from RSV infection was done as previously described [16]i.e.: the titre of cord, first and second samples for an individual were defined as TC, T1 and T2, respectively. For individuals with T1≥TC, all results for that individual were excluded, and for individuals with T2≥T1 the result for sample T2 was excluded. In addition, results from samples collected from cases after an infection was identified were excluded. Comparison of the estimated rate of decay was conducted using a two sample paired t-test. To further control for the difference in the estimated rate of decay between cases and controls within a match set, a linear regression model with an interaction effect between age and case/control was used.

The association between the concentration of maternal antibody titres and gestational age (measured by clinical evaluation or based on date of last menstruation) and birth weight (measured using a weighing scale at birth) was assessed. The odds of severe RSV disease in the first 6 months of life was determined, adjusting for the potential confounders of prematurity (i.e. gestational age < 37 weeks) and low birth weight (i.e. <2.5Kg) as categorical variables, using conditional logistic regression methods[28].

To ascertain exposure among controls, a defined cut off value was computed from the mean cord blood IgA log AU plus 3 standard deviations. Any of the 3 or 6 months serum samples with an IgA level above this cut off value were defined as exposed and those below this cut off as unexposed.

## Results

There was a total of 64 participants from the KBC study admitted with laboratory confirmed RSV-LRTI at the KCH in their first 6 months of life from April 2002 to 2007. Of the 64 individuals, 30(46.9%) participants had a stored cord blood and two 3 monthly follow up blood samples available. The median (interquartile) age was 3 (1–4) months for the cases at the first RSV infection. One case had *Haemophilus influenzae* bacteria isolated from blood culture while the rest had no significant bacterial pathogen. There was no statistically significant difference in RSV specific neutralizing titres in the samples screened over time (P = 0.2). The baseline characteristics between cases and controls were similar as shown in Table 1.

**Table 1:** Characteristics of study participants.

Characteristic	Cases (n %)	Controls (n %)	Total (n %)	P value
**Sample size**	30	60	90	
**No of samples**				
cord blood	30 (33.3%)	60 (66.7%)	90 (100%)	
3 month	26 (28.9%)	57 (63.3%)	83 (92.2%)	
6month	17 (18.9%)	44 (48.9%)	61 (67.8%)	
**Age in months at first infection for cases only**				
0–2	13 (23.7%)	26	39	
3–5	17 (30.9%)	34	51	
**Mean birthweight in Kilograms (Kgs)**	3(2.8–3.2)	2.8(2.7–3.0)		0.21
**Mean gestational age in weeks**	38.4(36.9–40.0)	38.4(37.5–39.3)		0.99
**Mean (95% CI) cord blood titre in infants admitted<6months [PRNT(log_2_)]**	10.7(10.3–11.0)	10.8(10.6–11.1)		0.4
**Mean (95% CI) cord blood titre in infants admitted<3months [PRNT(log_2_)]**	10.3(9.6–10.9)	10.7(10.2–11.1)		0.3
**Mean (95% CI) cord blood titre in infants admitted 3–6 months [PRNT(log_2_)]**	11.0(10.6–11.3)	11.0(10.6–11.4)		0.9
**Mean (95% CI) decay rates[PRNT(log_2_)/month ≤6months]**	-0.46(-.53 -.38)	-0.55(-0.58-.51)		0.01
**Mean (95% CI) decay rates[PRNT(log_2_)/month <3months]**	-0.46(-.57 -.35)	-0.55(-.60 -.50)		0.10

Gestational age data was available for 68% [62: 18 cases, 44 controls] of the participants, while 90% (81: 27 cases, 54 controls) had data on birth weight. The mean birth weight was 3.0Kg (95% CI 2.8–3.2) and 2.8 Kg (95% CI 2.7–3.0) for cases and controls, respectively, while the mean gestational ages of cases and controls were 38.4 weeks (95% CI 36.9–40.0) and 38.4 weeks (95% CI 37.5–39.3), respectively. Neither birth weight (P = 0.23, t = -1.20) nor gestational age (P = 0.99, t = 0.006) were statistically significantly different between cases and controls (Table 1).

Testing for RSV specific IgA antibody to ascertain exposure among controls was done on samples from 53 (88.3%) participants. Out of those tested, 16 (30%) showed evidence of exposure to RSV infection i.e., there was a rise in IgA antibody concentration in the follow up samples above the defined mean cut off value of 1.7 log AU. A further 30 of the controls showed a rise in IgA antibody concentration but non above the defined cut off value.

The distribution of log-transformed PRNT titres for cord blood samples for both cases and controls approximated to normal; with a mean concentration of 10.65 (95% Confidence interval, CI; 10.3–11.0, variance 0.97, median 10.64) for cases and 10.8 (95% CI 10.6–11.1, variance 1.1, median 10.9) for controls.

The difference in mean log_2_PRNT cord blood neutralising antibody titres between cases admitted within the first 6 months of life, and including their controls (30 cases and 60 controls) was not statistically significant (P = 0.4, t = -0.78). Similarly, there was not a statistically significant difference applying the Wilcoxon rank-sum (Mann-Whitney) test (P>0.05). Further analysis of the difference in mean log_2_PRNT cord blood antibody levels between cases [10.6 (95%CI 10.0–11.1)] and controls [11.1(95%CI 10.6–11.6)], where cases were matched to controls with confirmed evidence of exposure to RSV infection, showed no evidence of a difference between the two groups (P = 0.2).

**Fig 1:**
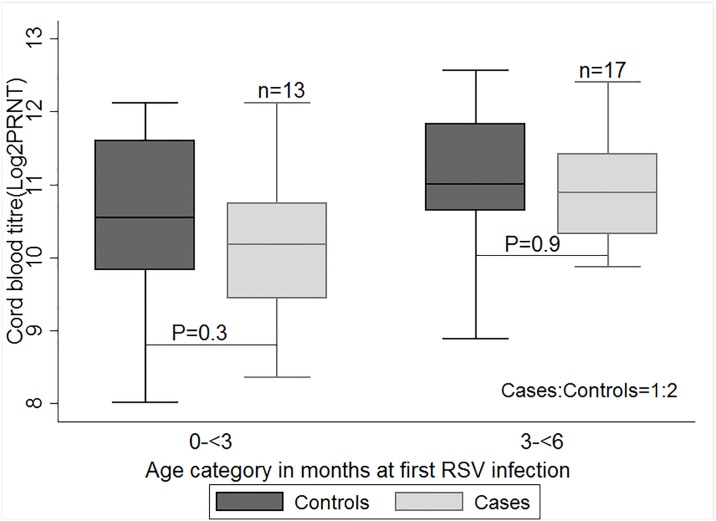
A box plot of the mean concentration of cord blood titres of cases and controls by age categories. Differences in the mean concentration of maternally transferred respiratory syncytial virus (RSV) specific antibodies (log_2_PRNT titres) from cord blood samples (with P values shown), between cases and controls at age categories in months of first RSV infection; 1–2 and 3–5. Cases were matched to controls in a ratio of 1:2. P values generated using a two sample paired t-test.

To investigate whether the protective effect of maternal neutralising antibody was related to age of the individual, and hence time from birth for antibody decay, we did a post-hoc analysis where the mean neutralising antibody titres in the cord bloods of cases and controls was stratified by the age of cases at which primary infection was identified (i.e. 0–2, 3–5 months) (Fig 1). No statistically significant difference in antibody levels between cases and controls was identified for any age category (Table 1). While cases in the 0–2 month age stratum had lower log_2_PRNT titres of RSV neutralising antibody in cord blood 10.3(SD: 1.1) than controls 10.7 (S.D:1.0), the difference was not statistically significant (P = 0.3, t = 1.03).

**Fig 2:**
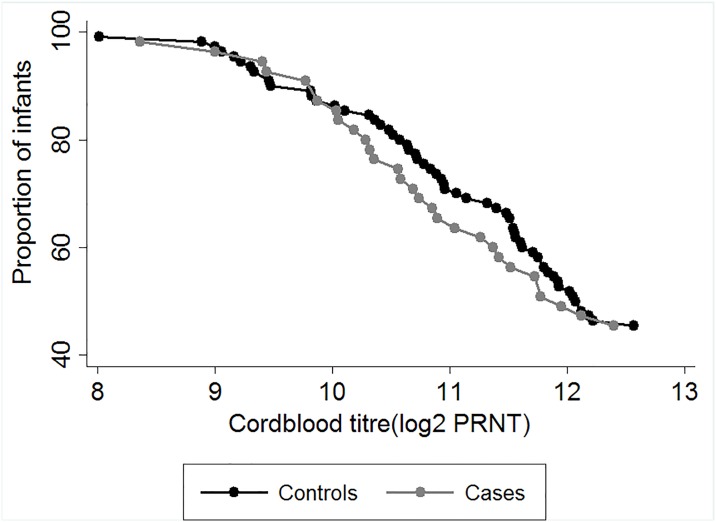
Reverse Cumulative Distribution plots of cord blood titres of cases and controls. The distribution of concentration of maternally transferred RSV specific antibodies (log_2_PRNT titres) from cord blood samples of infants born in Kilifi, Kenya. Grey symbols denote cord titres of cases while black symbols denote cord titres of controls.

To further investigate the relationship between level of RSV neutralising antibody in cord blood between cases and controls, a plot of reverse cumulative distribution of the neutralising titres was constructed (Fig 2). The graph shows considerable overlap in the distribution of cord blood RSV antibody titres among infants who became cases and those who did not, and thus, difficulty in defining a threshold of protection between the two groups.

**Fig 3:**
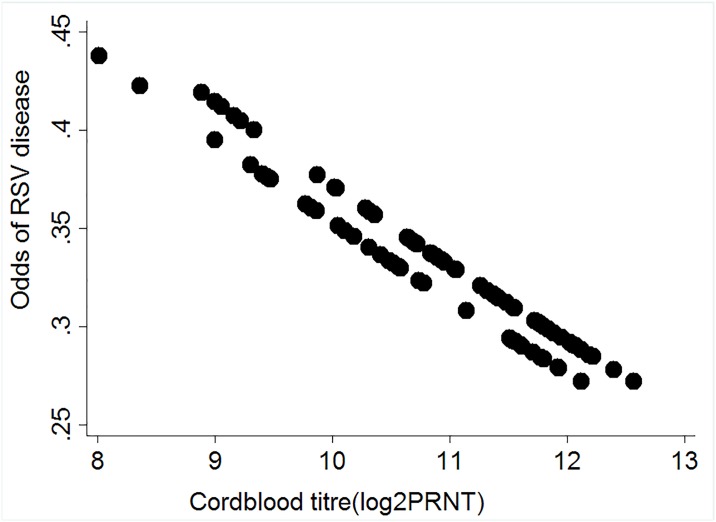
Relationship between Odds of RSV disease and the levels of Cord blood antibody titres. A scatter plot showing the odds of RSV associated hospitalization against maternally transferred RSV specific antibodies (log_2_PRNT titres) from cord blood samples of infants (both cases and controls) born in Kilifi, Kenya; 2002–2007. Black symbols denote individual cord titres for all 90 infants.

A plot of the odds of RSV disease against RSV neutralising antibody titres (Fig 3), by which to guide understanding of the role of antibody concentration in protection against RSV disease, suggests a decrease in odds of RSV disease with increase in cord blood neutralizing antibody titres; with a mean odds of 0.33 (95% CI 0.27–0.44) among all 90 infants.

We estimated a reduction in risk of RSV disease per log_2_PRNT unit increase in cord blood antibody titres using conditional logistic regression (though not statistically significant) of 33% (OR = 0.67, 95% CI 0.33–1.38; P = 0.2) and 5% (OR = 0.95, 95%CI 0.45–2.03; P = 0.9) among infant cases for whom their first RSV disease associated hospitalisation was between 0–2 and 3–5 months, respectively. The overall reduction in risk of RSV disease per log_2_PRNT unit increase in cord blood antibody titres in all infants was 21% (OR = 0.79, 95% CI 0.47–1.31; P = 0.4).

The log_2_PRNT antibody titre in cord blood showed an increase with increasing gestational age. However, adjusting for prematurity (i.e. gestational age < 37 weeks) in the conditional logistic regression analysis did not have an appreciable effect on the outcome (OR = 0.67, 95% CI 0.19–2.4; P = 0.5).

**Fig 4:**
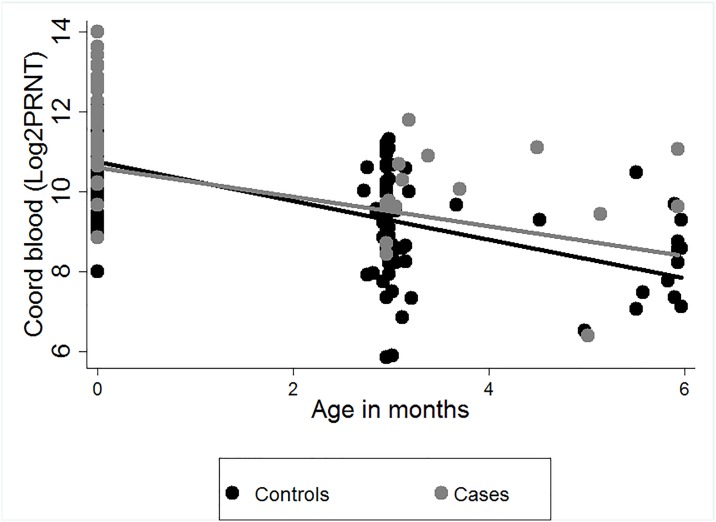
The estimated rate of decay of RSV specific antibodies from birth to 6 months of life among cases and controls. The estimated rate of decay of maternally transferred RSV specific antibodies (log_2_ transformed PRNT titres) over the first 6 months of life for infants (30 RSV cases and 60 controls) from a birth cohort, Kilifi, Kenya, with best fit linear decay models for samples from cases and controls. Grey symbols denote individual cord titres of cases while black symbols denote individual cord titres of controls.

The estimated rate of decay of RSV specific maternal antibodies (log_2_PRNT/month) for participant’s samples collected within 6 months of life was higher in controls -0.55(95% CI:-0.58 and-0.51) than in cases -0.46(95% CI:-0.53 and-0.38; P = 0.04, t = -2.09) (Fig 4). However, on estimating the rate of decay using samples collected from participants within the first 3 months of life, the rate of decay between the two groups was not significantly different (P = 0.1, t = -1.67). Including an interaction effect between age and case or control did not alter the results of the regression model using Huber sandwich estimator.

**Fig 5:**
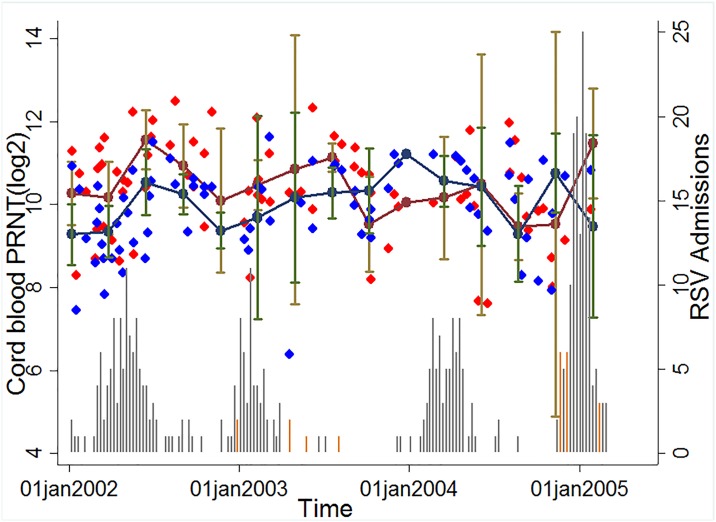
Dynamics of cord titres by time and transmission intensity. The Red diamond symbols denote individual cord titres by date of birth for RSV A2 strain, Blue diamond symbols denote individual cord titres by date of birth for RSV A Kilifi local strain, Maroon circle markers denote the mean cord titre by quarter (95% CI denoted by Brown whiskers) for RSV A2 strain, Navy blue circle markers denote the mean cord titre by quarter (95% CI denoted by Green whiskers) for RSV A Kilifi local strain. The Grey vertical bars (RSVA) and Orange vertical bars (RSV B) show the number of RSV IFAT positive paediatric severe or very severe pneumonia admissions to Kilifi County Hospital 2002–2005.

The distribution of cord neutralisation titres for RSV A2 and Kilifi local A strain against date of collection and overlaying with graphs for annual RSV epidemic in Kilifi for RSV A and B showed a similar trend indicative of cross protection, i.e., there was an increase in antibody levels among children born after an epidemic regardless of the RSV strain circulating within the population (Fig 5). However, the mean log_2_PRNT titres for A2 RSV strain were significantly higher (mean log_2_PRNT 10.6 [95% Confidence interval (CI) 10.3–10.7]) than those of the local RSV A circulating strain (mean log_2_PRNT 9.5 [95% CI 9.3–9.7]; (P<0.001). Similar results were observed for the comparison between RSV A2 and either the local circulating RSV B virus which had a mean log_2_PRNT of 10.3 [95% CI 10.1–10.5]; (P<0.01, t = 2.46*)* or the B860 RSV B strain(results not shown).

## Discussion

A key target group for RSV disease prevention is young infants under 6 months of age [4, 29], for whom the risk of severe disease is high. Previous studies have shown evidence that RSV specific maternal antibodies protect [9–11]. Consequently, maternal vaccination to boost the level of RSV-specific antibodies in pregnant women to extend the duration of protective antibodies in early infancy has been considered as a realistic approach [8, 9, 30]. However, the development of an effective vaccine would benefit from definitive information on the protective levels of RSV-specific neutralising antibodies. We undertook this study to describe the association between different levels of RSV specific maternal antibodies and the degree of protection against RSV disease among Kilifi infants.

In this study nested within a birth cohort from coastal Kenya, no significant difference in the mean concentration of cord RSV specific neutralizing antibodies between cases hospitalised with RSV and their controls was identified. Surveillance studies in Kilifi have found a high proportion of cases of RSV associated severe disease arising in the age group 0–2 months[24]; which is also an age group with highest titres of population level RSV specific maternal antibody. Surprisingly, we did not find any strong evidence of protection against severe RSV disease by neutralizing antibodies in this age group, although, the difference in RSV cord levels was slightly wider between cases and controls where cases had their first RSV associated hospitalization early in life (<3 months).

A similar lack of an overall protective effect by passively acquired maternal RSV neutralizing antibodies was previously observed by Bulkow in a study conducted among Alaskan native children [31]. An editorial to this study by Munoz and Glezen, suggested modifications to the analysis that might reveal a protective effect of maternal antibodies[32]. Among these suggestions were stratification of infants by age of infection, which was undertaken in the present study but did not reveal a statistically significant association.

To further understand why some infants get severe infection while others do not, we explored differences in rates of decay of maternally derived RSV specific antibody between cases and controls. This was based on the premise that infants with maternal antibodies which decay more rapidly are likely to get infected earlier in life. However, we did not see a differential in the rate of decay to support this hypothesis. Although we observed a significant difference in rate of decay between cases and controls within the first 6 months of life, this difference was unlikely to be of biological significance since the plots appeared similar for the 2 models. This observation is supported by previous analysis of the KBC cohort dataset where we did not find evidence of variation in the rate of decay with respect to the starting log_2_PRNT titre [16].

Transplacental transfer of RSV specific IgG antibodies occurs from the 28^th^ week of gestation and studies have shown this transfer to be very efficient [13]. In this study, cases and controls were equally distributed in gestational age. We also show that infants born premature have low levels of RSV specific maternal antibodies but this was unrelated to RSV disease. Nevertheless, it will be important to have alternative RSV disease preventive strategies for high risk infants especially those born premature, in the event there is a RSV maternal vaccine introduction to this population.

We observed from the RCD plots considerable overlap in the distribution of log_2_PRNT cord titres in those infants who became cases and those who did not. This suggests lack of a protective effect by RSV specific maternal antibodies in those infants who did not get RSV disease in this study. However, even though we cannot show a protective effect of RSV specific antibody within the range that is transferred naturally, this might not be the case for boosted levels associated with vaccination. The fact that high levels of intravenous immunoglobulin (IVIG) have been shown to be highly protective in other studies [33, 34], suggests that, there is a level of antibody above what is observed in natural exposure that protects.

Despite the overall lack of a protective effect, there is some evidence (though not statistically significant) of a degree of protection from severe disease conferred by RSV neutralizing antibodies. We estimated a reduction in odds of RSV disease associated hospitalization of 33% per unit increase in log_2_PRNT of RSV maternal neutralizing antibodies in age stratum 0–2 months. These results are suggestive that, rather than providing an all or nothing protection, RSV specific maternal antibodies confer a degree of protection against severe disease that decreases as the level declines.

Thus in summary, this study shows no effect of maternal antibodies at birth on the risk of RSV disease, no differential in the rate of decay of RSV specific antibody between cases and controls, nor a relationship between gestational age and risk of RSV disease. We therefore hypothesize that, development of RSV disease in this population might be associated with other underlying factors such as dose of infection, nutritional status, underlying immunity and environmental factors of which this study did not investigate. Our study also provides support for a recent conclusion of a randomised, blinded, controlled, dose- ranging study of a RSV recombinant fusion (F) nanoparticle vaccine in healthy women of childbearing age[35] which found that the immunity developed in response to wild-type RSV infection is insufficient in quality and quantity to confer high levels of protection. However, it remains plausible that a high level boosting of maternal antibody with high binding affinity will provide significant protection to the infant.

There are a number of possible limitations of the study that suggest caution in the interpretation of the results. In particular, limitations that might lead to concluding an absence of an association between cord RSV neutralising antibody level and protection from severe RSV disease, even when one existed. The number of cases of children admitted with severe RSV with cord blood samples was only 30, which diminishes the study power. It is also possible that controls differed from cases in the level and timing of exposure to RSV. We hoped to account for such exposure bias by closely matching cases to controls by date of birth and geographical location. We looked for evidence of exposure in the controls by testing sample residues for RSV specific IgA ELISA antibodies. A high proportion (87%) showed a rise in titre between sequential samples, although only for 30% was there a clear seroconversion. Interpretation of these data is difficult. Low or no rise in titre may not be conclusive of non-exposure given the sampling interval of 3 months which might allow infection, rise and subsequent fall to baseline of antibody levels, within the time period. Furthermore, maternal antibody might prevent infection and an acquired immune response. We undertook a sub-analysis of the data using cases matched to controls with IgA confirmed evidence of exposure which did not give different results from those observed from the overall analysis.

A further limitation is that we cannot exclude the possibility that the controls had been admitted with severe RSV to another hospital. However, all members of the KBC from which the cases and controls arise were recruited at the County hospital (KCH) with three monthly follow up visits to the KCH clinic, which strongly indicates that KCH would be the hospital to which sick children from the cohort would present. Of course, the matching of cases and controls may not remove differential exposure to RSV for which we have not accounted, such as family size, breast feeding, smoke exposure and underlying nutritional status or medical conditions.

Finally, we note that neutralising antibody titres are not internationally standardised and hence it is difficult to compare levels identified across different studies. Neutralising antibodies are directed to both the G and F proteins on the surface of the virus, but the proportion of each detected may depend upon the cell line chosen for the neutralisation assay [36]. This could result in underestimation of the protective levels of neutralising antibodies present. There is also the general question as to whether neutralising antibodies are the main or only mediator of maternal protection in infants.

## Conclusions

In this study in a birth cohort from a coastal Kenya population, there was no significant difference in cord antibody titres of RSV specific neutralising antibody between severe hospitalized RSV cases and a set of controls matched on date of birth and location without documented severe RSV. Based on the small sample size of infants hospitalized within the first 3 months of life, there is only limited evidence of a protective effect of maternal passively acquired antibody over the period 0–2 months of life. There was a suggestion of a relationship between the neutralisation titre at birth and degree of protection from severe RSV disease. It is possible that factors additional to specific neutralisation antibody levels determine susceptibility to RSV disease, and that levels of neutralizing antibody beyond the normal range derived from wild type exposure or of a different composition are required for protection, which it is hoped can be achieved by a maternal vaccine.

## Supporting Information

S1 Data Dataset. RSV dataset.csv
(CSV)

S2 Data Data dictionary. RSV data dictionary.xlsx
(XLSX)
